# Microelements as diagnostic markers of pancreatic cancer

**DOI:** 10.1186/1897-4287-13-S2-A2

**Published:** 2015-11-26

**Authors:** Marcin Lener, Anna Wiechowska-Kozłowska, Magdalena Muszyńska, Katarzyna Jaworska-Bieniek, Grzegorz Sukiennicki, Katarzyna Kaczmarek, Tomasz Gromowski, Wojciech Marciniak, Anna Jakubowska, Jan Lubiński

**Affiliations:** 1 Department of Genetics and Pathology, International Hereditary Cancer Center, Pomeranian Medical University, Szczecin, Poland; 2 Division of Health Care Ministry of Internal Affairs and Administration in Szczecin, Poland; 3 Read - Gene, S.A., Grzepnica, Poland

## Introduction

Pancreatic cancer is the eighth most commonly diagnosed cancer in the developed world and has one of the worst prognoses of any malignancy with 98% succumbing to their disease within 5 years. Little is known about the etiology of the disease despite significant new insights into the mutation signatures common this disease. Changes in microelements serum levels are reported in pancreatic cancer. In the current study we have examined the levels of Se, Cu, Fe, Zn and Mg in a moderately sized pancreatic cancer population and compared it to a healthy age-matched population.

## Material and methods

A total of 84 pancreatic cancer patients and 84 aged-matched healthy controls were enrolled in the study after providing informed consent. The patients with pancreatic cancer were enrolled to the study from the Hospital of the Ministry of Internal Affairs and Administration in Szczecin, Poland. For each pancreatic cancer patient included in this study an unaffected individual registered in International Hereditary Cancer Center, Pomeranian Medical University of Szczecin, was used as a control.

Each person enrolled in the study donated ~10 ml EDTA blood for sufficient serum to be isolated and examined for the elements Se, Cu, Fe, Zn and Mg. The level of Se, Cu, Fe, Zn and Mg, in the serum was determined by mass spectrometry Inductively Coupled Plasma Mass Spectrometry (Elan DRC-e, PerkinElmer).

## Results

**Table 1:** The correlation between serum Se level and the occurrence of pancreatic cancer.

Cancer site	Quartile	Se level (µg/l)	No. of cancer / controls	OR	p- value
**Pancreas**	**I**	29.87-55.40	37/5	1	-
	
	**II**	55.90-67.97	31/11	2.62	0.1635
	
	**III**	68.06-79.20	9/33	27.13	<0.0001
	
	**IV**	79.36-137.47	7/35	37	<0.0001

**Table 2:** The correlation between serum Cu level and the occurrence of pancreatic cancer.

Cancer site	Quartile	Cu level (µg/l)	No. of cancer / controls	OR	p- value
**Pancreas**	**I**	695.06-1023.95	9/32	1	-
	
	**II**	1025.88-1175.73	14/27	1.84	0.3256
	
	**III**	1179.38-1421.96	22/19	4.11	0.0058
	
	**IV**	1448.68-2901.35	37/4	32.8	<0.0001

**Table 3:** The correlation between serum Fe level and the occurrence of pancreatic cancer.

Cancer site	Quartile	Fe level (µg/l)	No. of cancer / controls	OR	p- value
**Pancreas**	**I**	138.88-668.75	29/12	1	-
	
	**II**	672.81-921.18	19/22	2.79	0.0429
	
	**III**	931.41-1245.91	13/29	5.39	0.0004
	
	**IV**	1247.89-1631.56	22/20	2.19	0.1151

**Table 4:** The correlation between serum Zn level and the occurrence of pancreatic cancer.

Cancer site	Quartile	Zn level (µg/l)	No. of cancer / controls	OR	p- value
**Pancreas**	**I**	306.12-760.79	23/18	1	-
	
	**II**	765.29-863.96	14/27	2.46	0.0752
	
	**III**	864.59-995.08	21/21	1.27	0.6620
	
	**IV**	995.84-1890.96	25/17	0.86	0.8257

**Table 5:** The correlation between serum Mg level and the occurrence of pancreatic cancer.

Cancer site	Quartile	Mg level (µg/l)	No. of cancer / controls	OR	p- value
**Pancreas**	**I**	11964.63-18616.02	25/16	1	-
	
	**II**	18673.87-20144.41	18/23	1.99	0.1843
	
	**III**	20274.88-21673.70	17/24	2.20	0.1215
	
	**IV**	21775.80-30676.08	22/19	1.34	0.6555

## Conclusions

1. There is a very strong correlation between the level of selenium, copper in serum and the occurrence of pancreatic cancers in the Polish population.

2. The Se, Cu level in serum may be a useful diagnostic tool of pancreatic cancer.

3. Further investigations are needed to determine if Se, Cu levels can be used in:

a. differential diagnosis between pancreatic cancer (PC) and non-malignant pancreatic lesions

b. monitoring of systemic treatment efficiency in PC

c. identification of prognostic factors of pancreatic tumors

d. identification of factor of PC causative

